# Idiopathic multicentric Castleman's disease: a clinicopathologic study in comparison with IgG4-related disease

**DOI:** 10.18632/oncotarget.24068

**Published:** 2018-01-09

**Authors:** Kyoko Otani, Dai Inoue, Kohei Fujikura, Takahiro Komori, Shiho Abe-Suzuki, Takuma Tajiri, Tomoo Itoh, Yoh Zen

**Affiliations:** ^1^ Department of Diagnostic Pathology, Kobe University Graduate School of Medicine, Kobe, Japan; ^2^ Department of Radiology, Kanazawa University Graduate School of Medicine, Kanazawa, Japan; ^3^ Department of Diagnostic Pathology, Tokai University Hachioji Hospital, Tokyo, Japan

**Keywords:** Castleman, IL-6, IgG4, autoimmune pancreatitis, pathology

## Abstract

The present study aimed to compare clinicopathologic features between idiopathic multicentric Castleman's disease (n=22) and IgG4-related disease (n=26). Histology was analyzed using lymph node and lung biopsies. The expression of IL-6 mRNA in tissue was also examined by *in situ* hybridization and real-time PCR. Patients with idiopathic multicentric Castleman's disease were significantly younger than those with IgG4-related disease (p<0.001). Splenomegaly was observed in only idiopathic multicentric Castleman's disease (p=0.002), while pancreatitis and sialo-dacryoadenitis were restricted to IgG4-related disease (both p<0.001). Serum IgG4 concentrations were commonly elevated at >135 mg/dL in both groups (p=0.270). However, the IgG4/IgG ratio in IgG4-related disease was significantly higher than that in Castleman's disease (p<0.001). Histologically, sheet-like plasmacytosis was highly characteristic of idiopathic multicentric Castleman's disease (p<0.001), while plasmacytic infiltration in IgG4-related disease was always associated with intervening lymphocytes. Similar to laboratory findings, the IgG4/IgG-positive plasma cell ratio, but not the IgG4-positive cell count, was significantly higher in IgG4-related disease (p=0.002). Amyloid-like hyalinized fibrosis was found in 6/8 lung biopsies (75%) of Castleman's disease. The over-expression of IL-6 mRNA was not confirmed in tissue samples of Castleman's disease by either *in situ* hybridization or quantitative real-time PCR. In conclusion, useful data for a differential diagnosis appear to be age, affected organs, the serum IgG4/IgG ratio, sheet-like plasmacytosis in biopsies, and the IgG4/IgG-positive cell ratio on immunostaining. Since IL-6 was not over-expressed in tissue of idiopathic multicentric Castleman's disease, IL-6 may be produced outside the affected organs, and circulating IL-6 may lead to lymphoplasmacytosis at nodal and extranodal sites.

## INTRODUCTION

Castleman's disease is a heterogenous entity typically presenting lymphadenopathy with characteristic histopathologic changes [1]. It is classified as unicentric or multicentric with the former showing lymphadenopathy restricted to a single anatomical site and indolent clinical courses [1, 2]. Multicentric Castleman's disease (MCD) is a more aggressive, polyclonal lymphoproliferative condition showing the enlargement of multiple lymph nodes and various systemic inflammatory manifestations (e.g., fever, elevated C-reactive protein [CRP], and hyperglobulinemia) [1, 3, 4]. In addition to lymph nodes, extranodal organs, particularly the lungs and kidneys, may be involved [5–10]. Another classification scheme of Castleman's disease is based on its association with human herpes virus 8 (HHV8) infection. Unicentric Castleman's disease is generally HHV8-negative, while up to 50% of MCD cases are associated with HHV8 [1–3]. By combining these two classification models, Castleman's disease is separated into HHV-8-negative unicentric disease, HHV8-positive MCD, and HHV8-negative “idiopathic” MCD (iMCD) [1]. Recent studies have also described MCD associated with thrombocytopenia, anasarca, fever, reticulin fibrosis/renal failure, and organomegaly (TAFRO) syndrome, which is another uncommon form of Castleman's disease [11, 12].

International consensus criteria were recently proposed for iMCD [13]. They are composed of 2 major and 11 minor criteria, with the 2 major and 2 out of the 11 minor items being required for a diagnosis. One major criterion is the confirmation of characteristic histologic features of the lymph nodes, while the other is the presence of lymphadenopathy in two or more nodal stations. Five microscopic findings listed in the major criterion are (1) regressed germinal centers, (2) follicular dendritic cell prominence, (3) vascularity, (4) hyperplastic germinal centers, and (5) plasmacytosis, with each finding being further graded into 0 to 3 [13]. In order to satisfy this histologic category, patients require grade 2 or 3 for either regressed germinal centers or plasmacytosis. Although serum IL-6 concentrations are commonly increased in patients with iMCD [1, 3], elevations in this inflammatory cytokine are a supportive finding, but not essential for a diagnosis in this proposed standard.

IgG4-related disease (IgG4-RD) is a systemic inflammatory disorder histologically characterized by plasma cell-rich, sclerosing inflammation potentially affecting various organs including the pancreas, salivary glands, lacrimal glands, bile duct, lungs, lymph nodes, kidneys, retroperitoneum, and aorta [14–20]. Since iMCD and IgG4-RD are both multi-organ disorders with similar histologic features of a plasma cell-rich inflammatory infiltrate, difficulties are associated with discriminating between the two conditions. However, this differential diagnosis is crucial for clinical management because IgG4-RD responds well to corticosteroid monotherapy, while patients with iMCD typically require IL-6 receptor blockade with tocilizumab to achieve remission [1, 14]. We have increasingly encountered cases in which a differential diagnosis between the two conditions is really challenging. This is particularly reinforced in the past several years, possibly because awareness of IgG4-RD is widespread and, thus, serum IgG4 concentrations are more commonly measured than before. Therefore, we were prompted to perform the present retrospective study in order to clarify how similar and dissimilar these two conditions are.

In the present study, we compared clinicopathologic features between iMCD and IgG4-RD. Since IgG4 is a biomarker for IgG4-RD, IgG4 elevations in tissue and blood were examined in both conditions using immunohistochemistry and serological tests, respectively. The expression of IL-6 mRNA in tissue samples was also analyzed using *in situ* hybridization and real-time PCR in order to elucidate the cellular origin of IL-6 production in iMCD and establish whether tissue examinations for IL-6 mRNA expression contribute to the diagnosis.

## RESULTS

### Case selection

This study was approved by the Ethics Committee of the Kobe University Graduate School of Medicine. A search of the pathology database at our institutes and affiliated hospitals identified 22 patients with iMCD. All tissue samples taken in iMCD cases were biopsies from either the lymph nodes or lungs. Therefore, 26 cases of IgG4-RD, in which lymph node or lung biopsies were available, were selected for comparison from our institutional database. Cases of iMCD were diagnosed during 16 years from January 2002 to December 2016, while the total number of IgG4-RD cases diagnosed at our institutes in the same period was more than 250 with a part of the cohort reported in a previous study [21]. Among the 22 cases of iMCD, 14 underwent lymph node biopsies, while the remaining 8 had lung biopsies (video-associated thoracoscopic biopsy, n=7; transbronchial biopsy, n=1). Similarly, lymph node samples were available in 10 cases of IgG4-RD and lung biopsies in the remaining 16 (transbronchial biopsy, n=10; video-associated thoracoscopic biopsy, n=5; percutaneous needle biopsy, n=1).

### Diagnostic criteria of iMCD and IgG4-RD

Since this study started before the diagnostic criteria for iMCD were proposed [13], no standardized diagnostic scheme for iMCD was available. Therefore, the diagnosis of iMCD was made by the combination of a plasma cell-rich inflammatory infiltrate in the lymph nodes or extranodal tissue, elevations in serological inflammatory markers (e.g., CRP), hyper-gammaglobulinemia, and an increased IL-6 concentration. Polyclonal plasmacytic infiltration to variable degrees was confirmed in all biopsy samples. Increased concentrations of CRP were observed in all (100%), hyper-IgG in 19 (86%), and IL-6 elevations in 19/20 cases tested (95%). When the proposed diagnostic criteria were retrospectively applied, 14 who had lymph node biopsies met the criteria, while the remaining 8 did not because of the lack of lymph node biopsy (an essential major criterion). In addition, 3 of the latter 8 cases did not have obvious lymphadenopathy on imaging at the initial presentation. However, we included the 3 cases in this study because their histologic features of lung biopsies and serological findings were basically similar to those of the other cases (more details described below). The possibility of HHV8-associated MCD was excluded by immunostaining for HHV8 using an anti-HHV8 antibody (clone 13B10; dilution 1:50; Leica Microsystems, Newcastle, UK). The polyclonal nature of plasmacytosis was also confirmed by *in situ* hybridization for immunoglobulin light chains (Ventana Medical Systems, Inc., Tucson, AZ).

The diagnosis of IgG4-RD was established based on the international consensus statement for IgG4-RD [22]. All patients had at least one biopsy or surgical specimen, the histologic features of which were consistent with IgG4-RD. In addition, serum IgG4 concentrations were elevated to greater than the upper limit of normal (>135 mg/dL) in all cases (100%). The involvement of at least one extranodal organ was confirmed in all cases, with two or more extranodal organs being affected in 17 (65%).

### Clinical features

Table 1 compares clinical features between the two groups. Patients with iMCD were significantly younger than those with IgG4-RD (p<0.001) with the youngest age being 19 years for iMCD and 44 years for IgG4-RD. The male-to-female ratio in IgG4-RD was slightly higher than that in iMCD (p=0.077). Although approximately 50% of cases in each group presented with mixed nodal and extranodal involvement, lymphadenopathy without other organ involvement was a manifestation only observed in iMCD (p=0.015). A wide range of organs were involved in either condition. Lymph node enlargement and lung manifestation were similarly observed in the two groups, possibly because IgG4-RD cases with nodal and/or pulmonary manifestations were selected for this study. Splenomegaly was the third most common manifestation in patients with iMCD (32%), but was not found in patients with IgG4-RD (p=0.002). Hepatomegaly was also restricted to the iMCD group, but did not reach a significant difference. In contrast, pancreatitis and sialo-dacryoadenitis were observed in approximately 50% of patients with IgG4-RD, but none of the iMCD cases (both p<0.001).

**Table 1:** Comparison of clinical features between iMCD and IgG4-RD

	iMCD (n=22)	IgG4-RD (n=26)	p-value
Age			
Mean ± SD (years)	53 ± 13	69 ± 10	<0.001
<50 years (n, %)	8 (36%)	2 (8%)	0.029
Gender (male %)	45%	73%	0.077
Organ involvement			
Nodal	5 (23%)	0	0.015
Mixed nodal and extranodal	14 (63%)	14 (54%)	0.565
Extranodal	3 (14%)	12 (46%)	0.027
Affected organs (n, %)			
Lymph nodes	19 (86%)	14 (54%)	0.027
Lungs	15 (68%)	18 (69%)	1.000
Spleen (splenomegaly)	7 (32%)	0	0.002
Liver (hepatomegaly)	3 (14%)	0	0.089
Kidney	2 (9%)	8 (31%)	0.085
Ureter/renal pelvis	1 (5%)	2 (8%)	1.000
Retroperitoneum	1 (5%)	3 (12%)	0.614
Paravertebral mass	1 (5%)	1 (4%)	1.000
Prostate	0	1 (4%)	1.000
Pericardium	0	2 (8%)	0.493
Aorta (periaortitis)	0	3 (12%)	0.239
Bile duct	0	5 (19%)	0.054
Pancreas	0	12 (46%)	<0.001
Salivary/lacrimal glands	0	14 (54%)	<0.001

SD, standard deviation.

Systemic symptoms, such as fever, weight loss, and fatigue were observed in only iMCD cases (8/22, 36%), one of which also had pleural effusion and ascites. As shown in Table 2, IgG concentrations in iMCD were significantly higher than those in IgG4-RD (p<0.001). Except for two cases of iMCD, serum IgG4 concentrations were elevated to greater than the upper limit of normal (135 mg/dL) in all cases tested, with no significant difference in IgG4 concentrations between the two groups (p=0.270). The IgG4/IgG ratio (normal range <5%) in IgG4-RD was significantly higher than that in iMCD, with the ratio being elevated >10% in 25/26 cases (96%) of IgG4-RD and 5/16 cases (31%) of iMCD (p<0.001).

**Table 2:** Comparison of laboratory findings between iMCD and IgG4-RD

	iMCD (n=22)	IgG4-RD (n=26)	p-value
IgG			
Concentrations (mg/dL)^*^	4,782 (811-31,313)	2,098 (1,150-4,661)	<0.001
>1x ULN (n, %)	19 (86%)	20 (77%)	0.478
>2x ULN (n, %)	16 (73%)	5 (19%)	<0.001
IgG4			
Concentrations (mg/dL)^*^	483 (50-1,230) ^†^	611 (146-2,140)	0.270
>1x ULN (n, %)	14 (88%)^†^	26 (100%)	0.139
>2x ULN (n, %)	12 (75%)^†^	21 (81%)	0.956
IgG4/IgG ratio			
Ratio (%)	8.6 (1.5-23.6) ^†^	24.1 (8.7-59.0)	<0.001
>5% (n, %)	12 (75%)^†^	26 (100%)	0.016
>10% (n, %)	5 (31%)^†^	25 (96%)	<0.001
CRP			
Concentrations (mg/dL)^*^	6.7 (1-50.4)	0.1 (0-1.25)	<0.001
>1x ULN (n, %)	22 (100%)	5 (19%)	<0.001
>5x ULN (n, %)	20 (91%)	0	<0.001
Hemoglobin (g/dL)			
Concentration (g/dL)^*^	9.9 (6.6-13.4)	13.6 (9.6-15.5)	<0.001
<1x LLN (n, %)	21 (95%)	10 (38%)	<0.001
IL-6 (pg/mL)			
Concentration (pg/mL)^*^	21.6 (4-76)^‡^	NA	NA
>1x ULN (n, %)	19 (95%)^‡^	NA	NA

ULN, the upper limit of normal; LLN, the lower limit of normal; ^*^, data are shown as a median (range); ^†^, data available in 16 cases; ^‡^, data available in 20 cases. Normal ranges: IgG, 870-1,700 mg/dL; IgG4, <135 mg/dL; CRP, 0-0.3 mg/dL; hemoglobin, 11.8-15.0 g/dL (female), 13.6-17.0 g/dL (male); IL-6, 0-4 pg/mL.

In 3 patients with iMCD, lymphadenopathy was not obvious on imaging, and, thus, did not meet the proposed diagnostic criteria for iMCD [13]. Their clinicopathologic features are summarized in Table 3. Although lymphadenopathy was not confirmed at the initial presentation, lymph nodes had gradually enlarged and became >1 cm in two cases during follow-up periods. Two of them were symptom-free and were incidentally found to have abnormalities in the lungs on imaging during a routine medical check-up. The remainder, who presented with weight loss, had manifestations in the lungs, renal pelvis, and ureter, but lacked lymph node enlargement.

**Table 3:** Clinicopathologic features of iMCD cases that initially presented without lymphadenopathy

Case	Age/sex	Affected organs	Laboratory data			Treatment and clinical course	Pathologic findings			
			CRP (mg/dL)	Hb (g/dL)	IL-6 (pg/mL)		Sheet-like plasmacytosis	Hyalinized fibrosis	Lymphoid follicles	IgG4/IgG (%)
1	67M	Lung, renal pelvis, and ureter	1.0	10.9	33.6	He declined treatment. The disease has been gradually progressed in the 10-year follow-up period. Mediastinal lymph nodes also have enlarged more than 1 cm.	Present	Present	Absent	39
2	46M	Lung	2.3	13.4	32.4	Treated with prednisolone, but limited responses obtained. Abdominal and paraaortic lymph nodes have gradually enlarged more than 1 cm during the 13-year follow-up period.	Present	Present	Present	33
3	37F	Lung and mild splenomegaly	1.5	11.5	9.4	Initially treated with prednisolone. Additional tocilizumab led to clinical improvement. Since it was stopped due to adverse effects, the disease has gradually deteriorated. No lymph node enlargement >1 cm is observed so far (12 years).	Present	Present	Present	46

CRP, C-reactive protein; Hb, hemoglobin; PSL, prednisolone; IgG4/IgG, IgG4/IgG-positive plasma cells ratio. Normal ranges: IL-6, 0-4 pg/mL; CRP, 0-0.3 mg/dL; Hb, 11.8-15.0 g/dL (female), 13.6-17.0 g/dL (male).

Of the 22 patients with iMCD, 6 (27%) received corticosteroid monotherapy, but showed only partial responses. Additional tacrolimus was given to one patient, but remission was not obtained. Of 12 (55%) patients who were treated with tocilizumab, 10 achieved clinical remission, while another case continued to have general malaise. The remaining patient initially treated with corticosteroid also responded to tocilizumab; however, the IL-6 blockage therapy was stopped due to adverse effects (case 3 in Table 3). Three (14%) patients were followed-up without treatment because of the lack of symptoms. Treatment data were not available for the remaining consultation case (4%). In contrast, all but two patients (92%) with IgG4-RD responded well to corticosteroids, leading to clinical remission, while the remaining two (8%) with no symptoms did not receive treatment and no progression was observed at the time of writing this manuscript.

### Lymph node pathology

**Figure 1:**
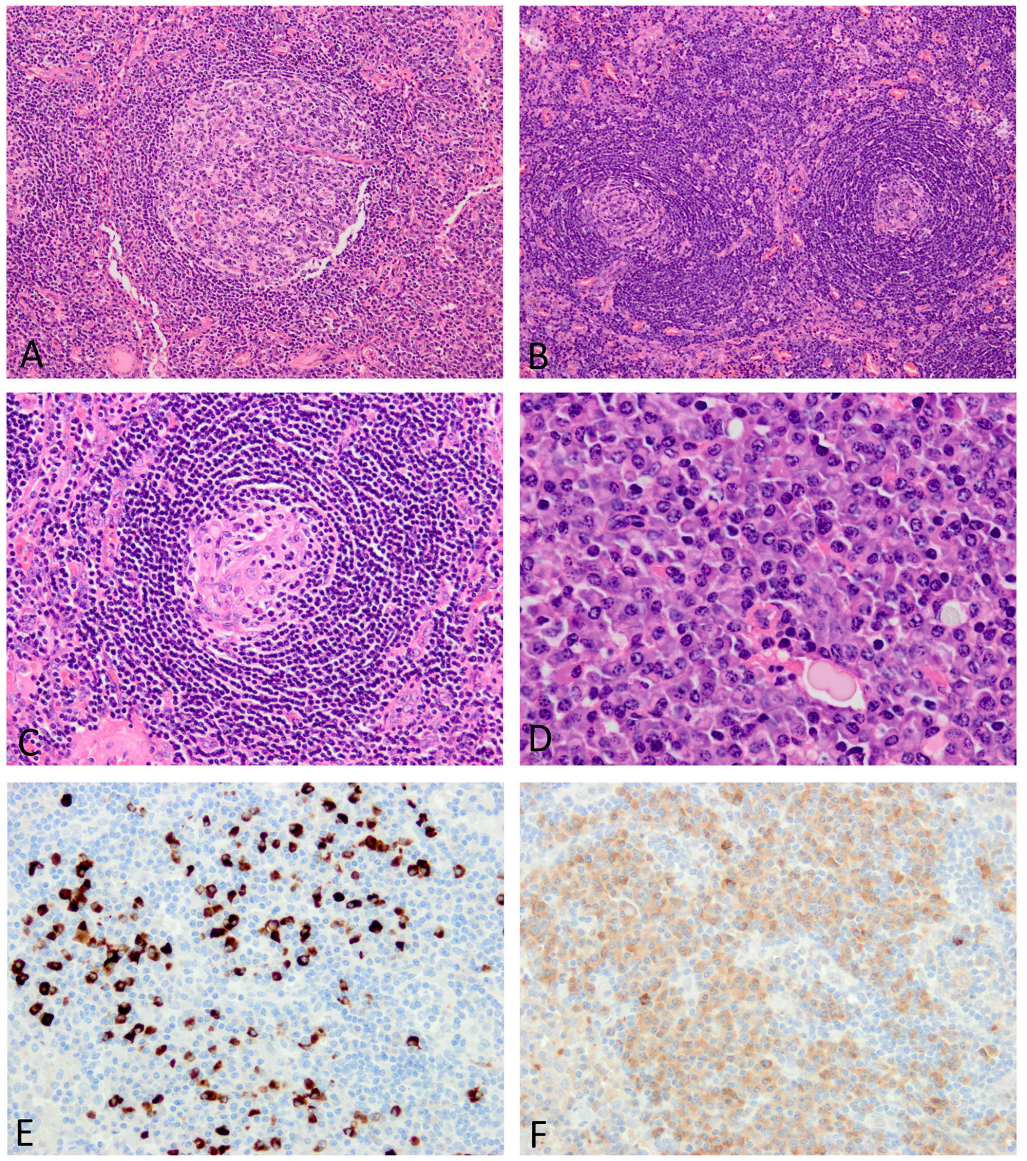
Histopathologic features of lymph nodes involved in iMCD **(A)** The lymphoid follicle is hyperplastic. **(B)** The mantle zone is expanded with a laminated appearance, while germinal centers are regressed. **(C)** Follicular dendritic cells are prominent with a small number of lymphocytes in the germinal center. **(D)** The interfollicular area shows sheet-like plasmacytosis. **(E)** Many IgG4-positive plasma cells are observed (IgG4 immunostaining). **(F)** IgG-positive plasma cells also increase in number with an IgG4/IgG-positive plasma cell ratio <40% (IgG immunostaining). Images E and F were taken at almost the same fields.

Based on morphologic changes in lymph node biopsies, 10/14 cases of iMCD were categorized as the plasmacytic type with prominent plasmacytosis, while 2 were the hypervascular type characterized by regressed germinal centers with follicular dendritic cell predominance (Figure 1). The remaining 2 were of the mixed type.

**Figure 2:**
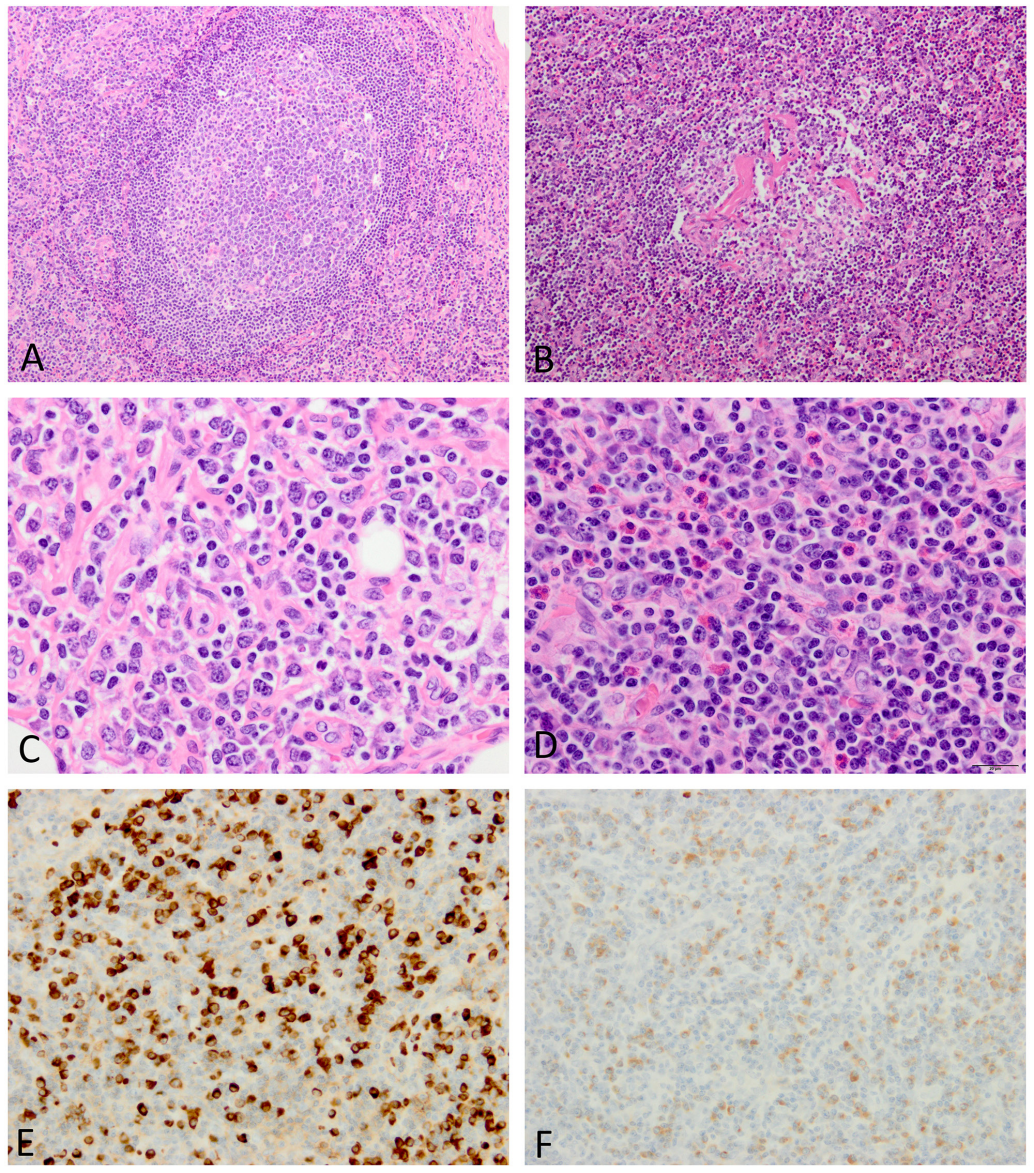
Histopathologic features of lymph nodes involved in IgG4-RD **(A)** The lymphoid follicle is hyperplastic. **(B)** Blood vessels in the germinal center are hyalinized. **(C)** Plasmacytic infiltration is associated with lymphocytes in between. **(D)** Occasional eosinophils are also observed. **(E)** Many IgG4-positive plasma cells are diffusely present (IgG4 immunostaining). **(F)** The IgG4/IgG-positive plasma cell ratio is increased to >40% (IgG immunostaining). Images E and F were taken at almost the same fields.

The presence of each histologic finding (grade 2 or 3) listed in the major diagnostic criteria for iMCD was compared between iMCD and IgG4-RD (Table 4). Follicular dendritic cell prominence, vascularity, and hyperplastic germinal centers were similarly observed in both conditions (Figures 1A-1C and 2A, 2B). The two histologic findings (regressed germinal centers and plasmacytosis), either of which is essential for the diagnosis of iMCD, were more commonly observed in iMCD than in IgG4-RD. This was particularly evident when only grade 3 plasmacytosis was counted, with sheet-like plasmacytosis being observed in 11/14 cases (79%) of iMCD, but none of IgG4-RD (p<0.001; Figure 1D). Although 7 cases of IgG4-RD showed grade 2 plasmacytosis, small and large lymphocytes were also present among plasma cells, the appearance of which was still insufficient for grade 3 plasmacytosis (Figure 2C). Eosinophilic infiltration was significantly more common in IgG4-RD than in iMCD (p=0.020) (Figure 2D). No fibrosis was observed in any lymph node biopsies examined in this study.

**Table 4:** Comparison of lymph node pathology between iMCD and IgG4-RD

	iMCD (n=14)	IgG4-RD (n=10)	p-value
Findings in iMCD diagnostic criteria (n, %)			
Regressed germinal centers (G2-3)	4 (29%)	1 (10%)	0.358
Follicular dendritic cell prominence (G2-3)	4 (29%)	3 (30%)	1.000
Vascularity (G2-3)	11 (79%)	6 (60%)	0.393
Hyperplastic germinal centers (G2-3)	6 (43%)	8 (80%)	0.104
Plasmacytosis (G2-3)	12 (86%)	7 (70%)	0.615
Plasmacytosis (G3)	11 (79%)	0	<0.001
Other findings (n, %)			
Eosinophil infiltration (>10 cells/hpf)	0	4 (40%)	0.020
Neutrophil infiltration (>10 cells/hpf)	0	0	
Broadened laminated mantle zone	5 (36%)	1 (10%)	0.341
Immunohistochemistry			
IgG4+ plasma cells			
Positive cell count (median, range)	247.5 (4-636)	236 (23-370)	0.953
>10 cells/hpf (n, %)	13 (93%)	10 (100%)	1.000
>50 cells/hpf (n, %)	12 (86%)	9 (90%)	1.000
IgG4+/IgG+ plasma cell ratio			
Ratio (median, range)	36.7 (2.1-71.5)	68.3 (43.6-94.9)	0.002
>40% (n, %)	5 (36%)	10 (100%)	0.002
>70% (n, %)	2 (14%)	4 (40%)	0.192

G2, grade 2; G3, grade 3.

In immunohistochemistry, IgG4-positive plasma cell counts did not differ between the two conditions, with IgG4-positive plasma cells >50 cells/hpf being commonly observed in both (Figure 1E and 2E). In contrast, the IgG4/IgG-positive plasma cell ratio in IgG4-RD was significantly higher than that in iMCD (p=0.002) (Figures 1E 1F and 2E, 2F). IgG4/IgG ratios were elevated to >40% in all cases of IgG4-RD, but only 5/14 (36%) cases of iMCD (p=0.002) (Table 4).

### Lung pathology

**Figure 3:**
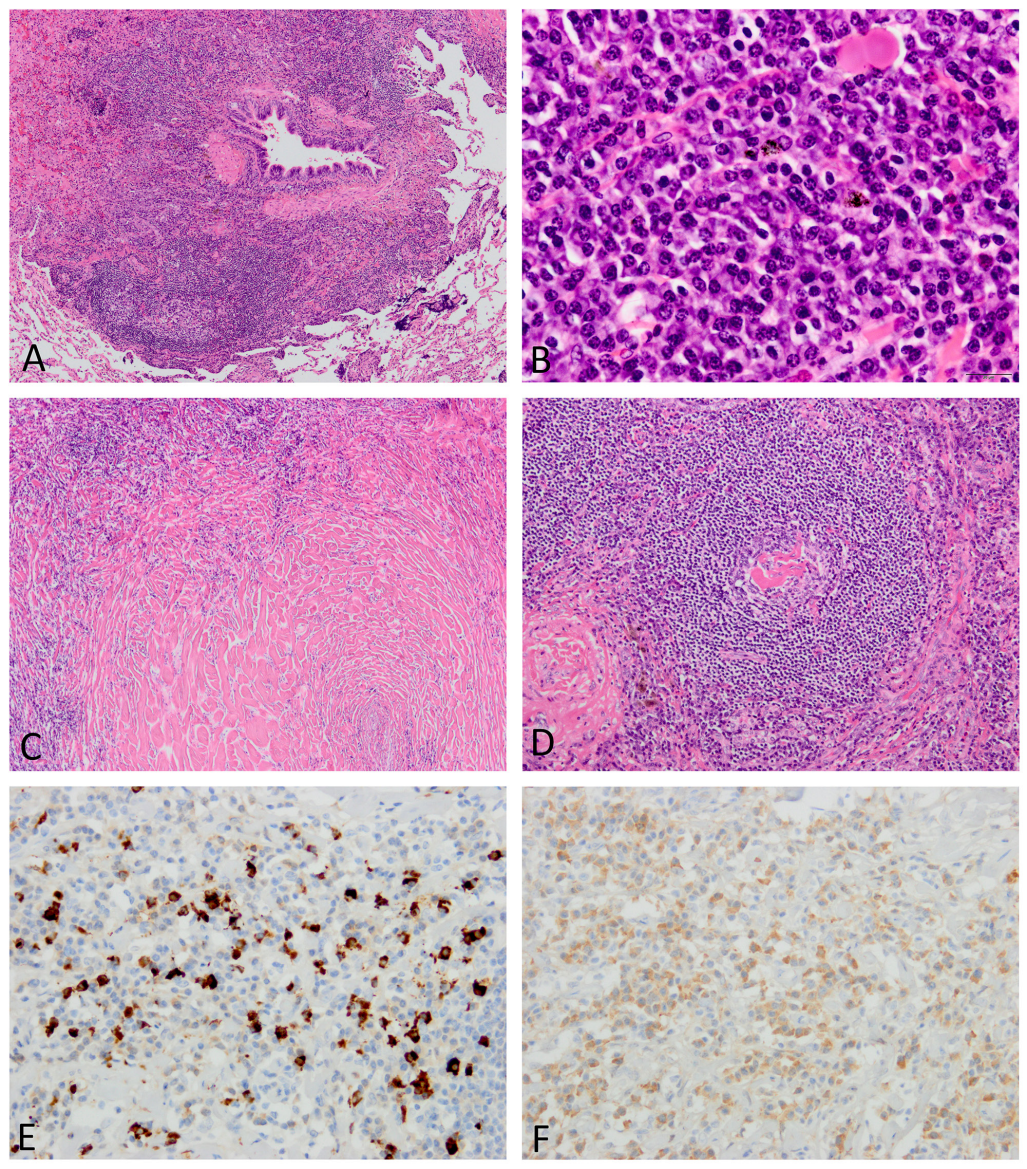
Histopathologic features of lungs involved in iMCD **(A)** A dense inflammatory infiltrate is observed around the bronchiole. **(B)** Many plasma cells are arranged in sheets with almost no intervening lymphocytes. **(C)** Amyloid-like hyalinizing fibrosis is broadly observed. **(D)** The lymphoid follicle contains a regressed germinal center with hyalinized blood vessels. **(E)** Many IgG4-positive plasma cells are identified (IgG4 immunostaining). **(F)** The ratio of IgG4/IgG-positive plasma cells are approximately 40% (IgG immunostaining). Images E and F were taken at almost the same fields.

**Figure 4:**
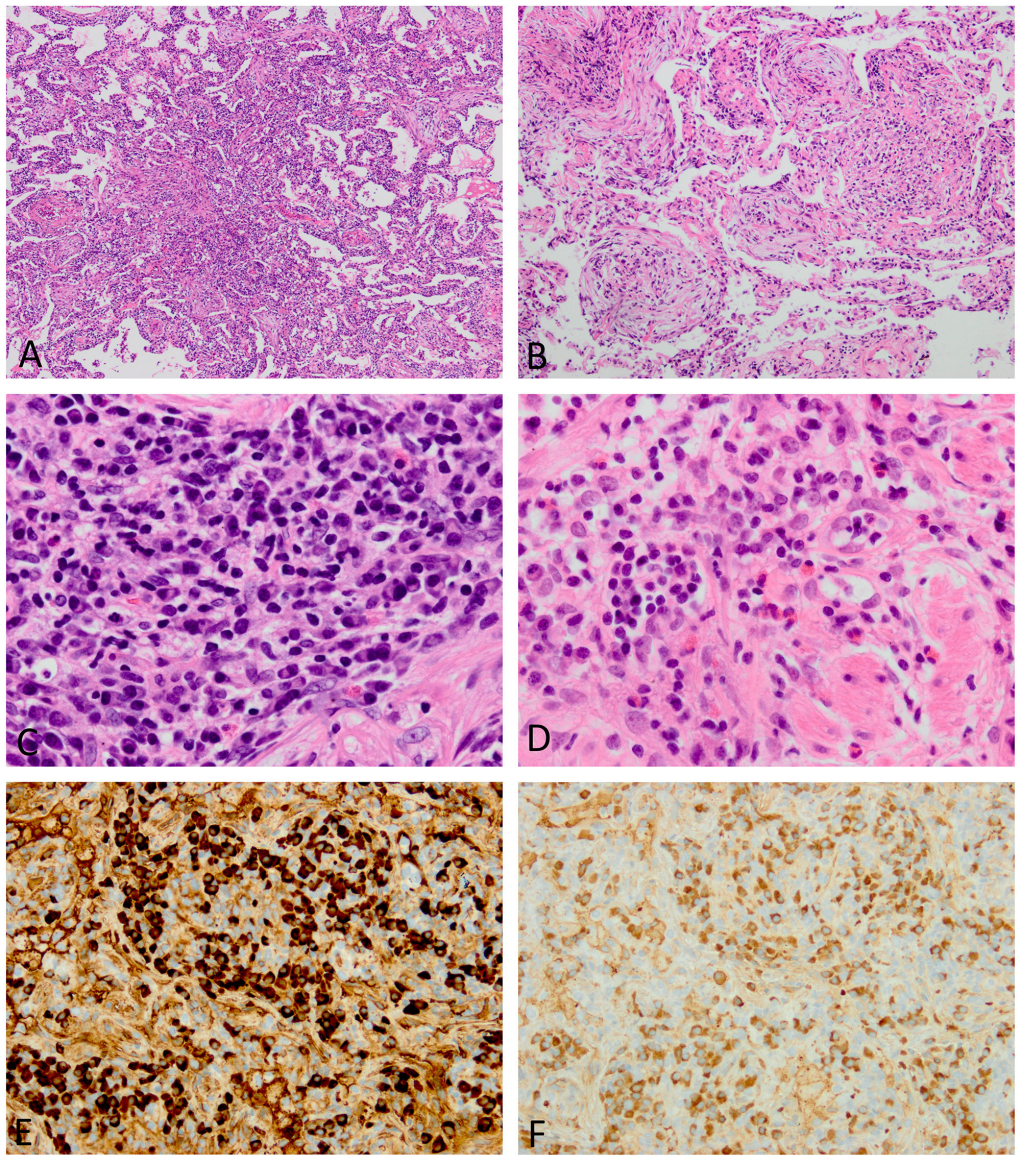
Histopathologic features of lungs involved in IgG4-RD **(A)** The inflammatory process infiltrates the alveolar interstitium. **(B)** An organizing pneumonia pattern is focally seen. **(C)** Although many plasma cells are observed, intervening lymphocytes are also present. **(D)** Occasional eosinophils are identified. **(E and F)** The absolute number of IgG4-positive plasma cells and ratio of IgG4/IgG-positive plasma cells are both increased (E, IgG4 immunostaining; F, IgG immunostaining). Images E and F were taken at almost the same fields.

Both conditions showed extensive inflammatory extension along intrapulmonary connective tissue, including bronchovascular bundles, the alveolar interstitium, interlobular septa, and pleura (Figures 3A and 4A). Areas of exaggerated inflammation often created nodular and mass lesions. Bronchovascular inflammatory extension and nodule formation were common in both conditions, while diffuse involvement in the alveolar interstitium resembling interstitial pneumonia was observed in 6/16 cases (38%) of IgG4-RD, but none of iMCD (Table 5). Three out of five iMCD cases with a predominantly nodular appearance also had peribronchovascular inflammatory extension elsewhere in the specimens. An organizing pneumonia pattern was focally observed in 4 cases of IgG4-RD (Figure 4B).

**Table 5:** Comparison of lung pathology between iMCD and IgG4-RD

	iMCD (n=8)	IgG4-RD (n=16)	p-value
Distribution pattern (n, %)			
Bronchovascular	3 (38%)	6 (38%)	0.655
Alveolar interstitial	0	6 (38%)	0.134
Nodular	5 (63%)	4 (25%)	0.180
Inflammatory cell infiltration (n, %)			
Sheet-like plasmacytosis	7 (88%)	1 (6%)	<0.001
Eosinophils (>10 cells/hpf)	2 (25%)	8 (50%)	0.388
Neutrophils (>10 cells/hpf)	1 (13%)	1 (6%)	1.000
Fibrosis pattern (n, %)			
Storiform fibrosis	0	2 (13%)	0.536
Hyalinized fibrosis	6 (75%)	0	<0.001
Obliterative phlebitis (n, %)	0	2 (13%)	0.536
Lymphoid follicles (n, %)	6 (75%)	4 (25%)	0.032
Regressed germinal centers (n, %)	1 (13%)	0	0.718
Follicular dendritic cell prominence (n, %)	2 (25%)	0	0.192
Immunohistochemistry			
IgG4+ plasma cells			
Positive cell count (median, range)	113 (62-319)	104 (34-611)	0.760
>10 cells/hpf (n, %)	8 (100%)	16 (100%)	Identical
>50 cells/hpf (n, %)	8 (100%)	13 (81%)	0.526
IgG4+/IgG+ plasma cell ratio			
Ratio (median, range)	35.7 (6.3-64.6)	79.4 (27.4-99.3)	0.002
>40% (n, %)	3 (38%)	15 (94%)	0.007
>70% (n, %)	0	9 (56%)	0.010

Similar to the lymph nodes, the lungs involved in iMCD often showed sheet-like plasmacytosis corresponding to grade 3 in the iMCD diagnostic criteria for lymph node biopsies (7/8 cases, 88%) (Figure 3B). Plasmacytic infiltration in IgG4-RD was always associated with intervening lymphocytes, and, thus, the appearance was still short of “sheet-like” plasmacytosis (Figure 4C). Eosinophilic infiltration was slightly more common in IgG4-RD, but did not reach a significant difference (50% vs. 25%, p=0.388) (Figure 4D). Storiform fibrosis was present in 2/16 cases (13%) of IgG4-RD, both having solid nodular lesions. Obliterative phlebitis, another characteristic finding of IgG4-RD [18, 22], was identified in only IgG4-RD (2/16 cases, 13%). Interestingly, 6/8 cases of iMCD had extensive hyalinized fibrosis (Figure 3C), the appearance of which somewhat resembled amyloidosis on H&E-stained sections; however, direct fast scarlet staining for amyloid was negative. Lymphoid follicle formation was significantly more common in iMCD (p=0.032), with regressed germinal centers and follicular dendritic cell prominence also being observed in 1 and 2 cases, respectively (Figure 3D).

Similar to lymph node biopsies, no significant difference was observed in IgG4-positive plasma cell counts between the two conditions, while the IgG4/IgG-positive plasma cell ratio was significantly higher in IgG4-RD (p=0.002) (Figures 3E, 3F and 4E, 4F). Elevated IgG4/IgG ratios >70% were noted in 9/16 cases (56%) of IgG4-RD, but none of iMCD (p=0.010) (Table 5).

### IL-6 mRNA *in situ* hybridization

**Figure 5:**
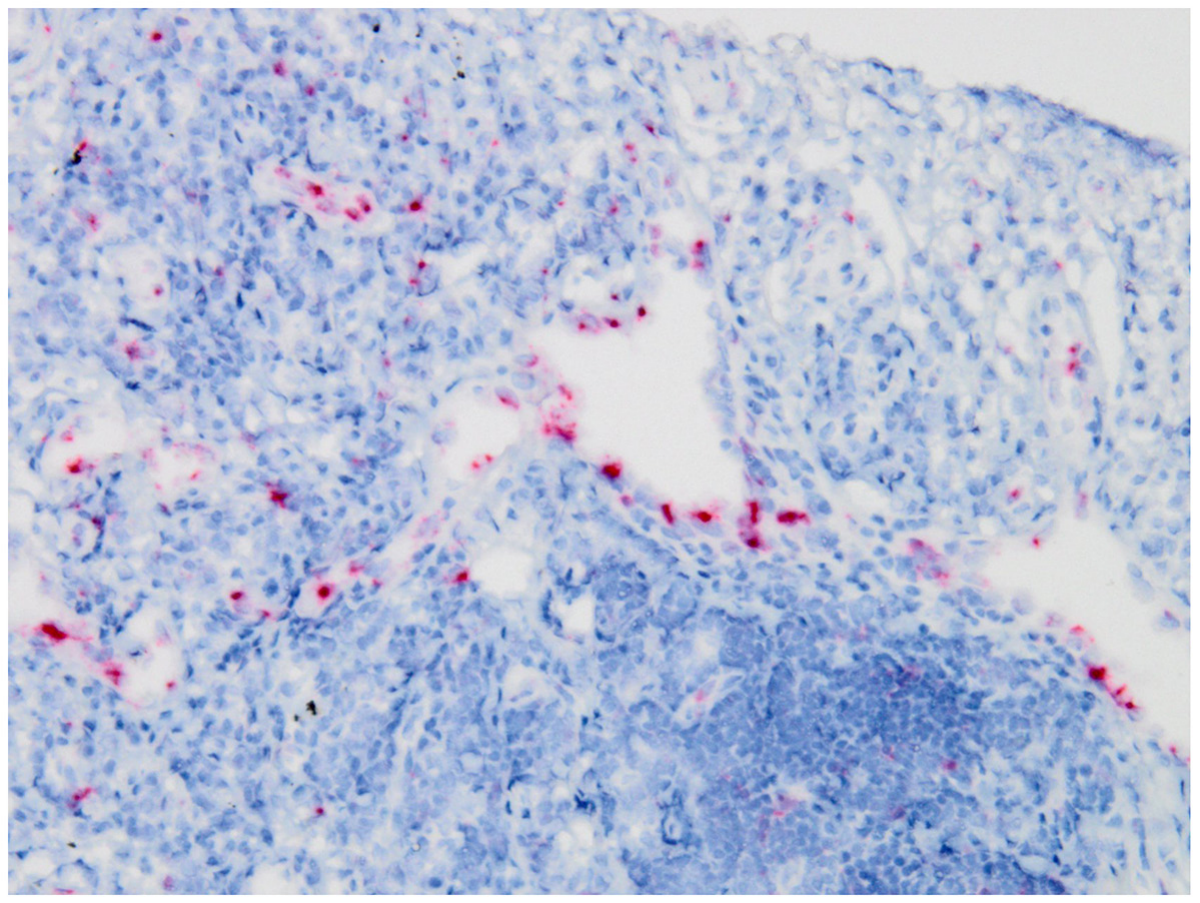
*In situ* hybridization for IL-6 mRNA in a lung biopsy of iMCD Positive signals are mainly observed in endothelial cells.

*In situ* hybridization was performed on all lymph node and lung biopsies of iMCD in order to elucidate which cells express IL-6 in iMCD. A single lung specimen was found to have a small focus showing positive signals in the endothelial cells of small vessels (Figure 5). However, the remaining cases appeared to be entirely negative.

### Quantitative real-time PCR

Since IL-6 expression was not obvious on *in situ* hybridization, real-time PCR for IL-6 mRNA was also conducted to further confirm that IL-6 is not overexpressed on site. Cases of non-specific reactive lymphadenopathy (n=5), other lung diseases (n=5), and normal lungs (n=5) were used as controls. The lung disease controls consisted of one case each of bone marrow transplantation-associated organizing pneumonia, rheumatoid arthritis-related interstitial pneumonia, aspergillosis, bronchopneumonia, and extranodal marginal zone B-cell lymphoma of mucosa-associated lymphoid tissue. Normal lung tissue was obtained from unremarkable background parenchyma in bullectomy specimens.

**Figure 6:**
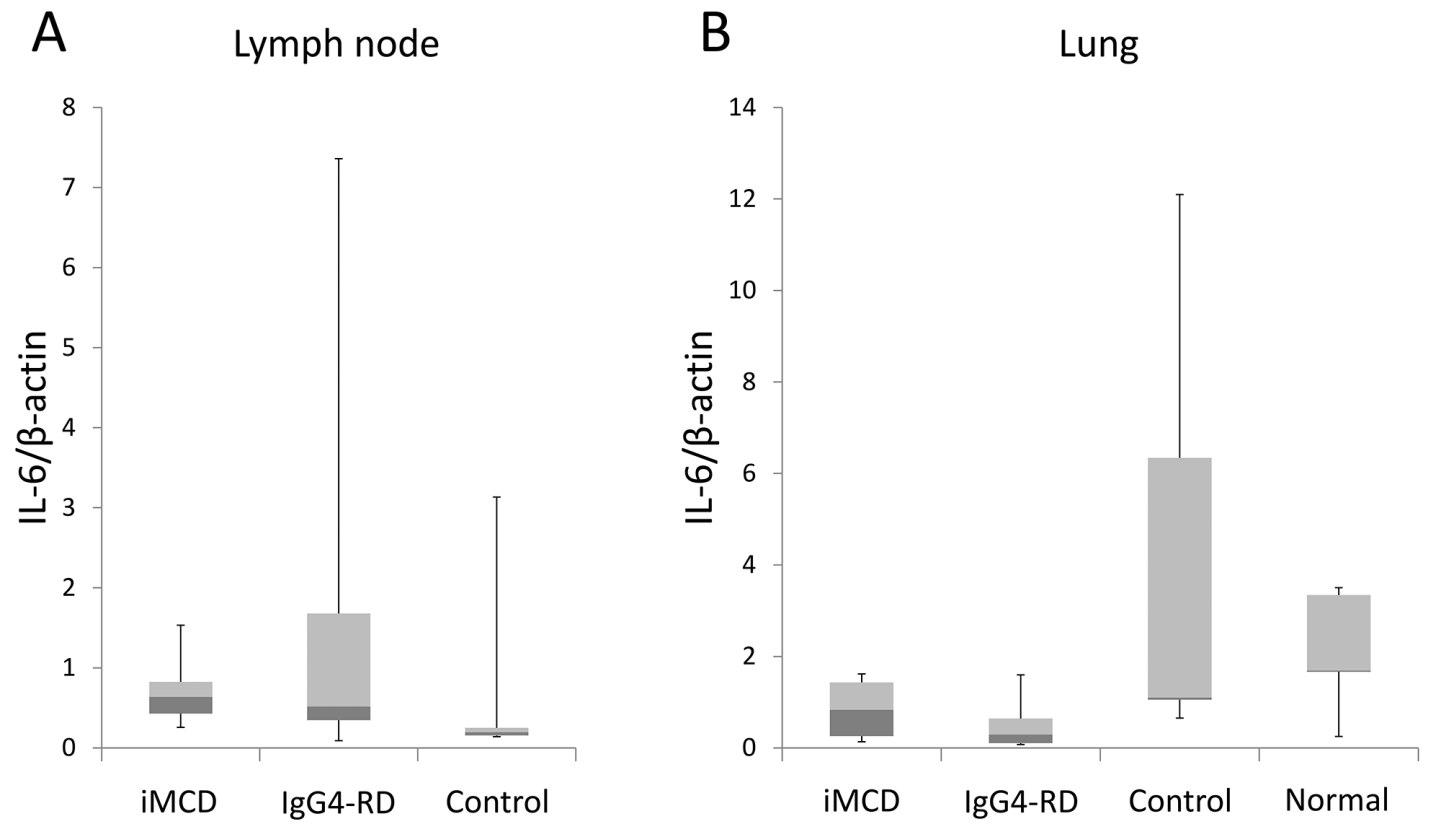
Results of quantitative real-time PCR for IL-6 No significant increase in IL-6 expression is observed in lymph node **(A)** or lung biopsies **(B)**. Data are shown as a median (lines across the boxes) and range. Boxes represent interquartile ranges.

In lymph node biopsies, no significant difference was observed in IL-6 expression values among iMCD, IgG4-RD, and disease controls (p=0.165, Figure 6A). In lung samples, IL-6/β-actin mRNA ratios were also similar between iMCD and IgG4-RD, and their values were slightly lower than those in disease controls and normal lung tissue (Figure 6B). The Kruskal-Wallis test among the four groups suggested a significant difference (p=0.032); however, no significant difference was confirmed between any two-group comparisons by the post-hoc analysis.

## DISCUSSION

The present study identified differences in clinical presentation, laboratory findings, and histopathologic features between iMCD and IgG4-RD. These parameters will help us to discriminate these conditions in clinical practice. Age appears to be important because iMCD more commonly developed in patients younger than 50 years than IgG4-RD. Affected organs overlapped between the two conditions. However, the presence of pancreatitis or sialo-dacryoadenitis suggests IgG4-RD more likely, while hepatosplenomegaly is a manifestation favoring iMCD. One of the interesting results of the present study was that serum IgG4 elevations were not useful for discriminating between the two conditions. This result also underscores the differential diagnosis of these two conditions being challenging based on serological findings alone. The present results also suggested that the IgG4/IgG ratio is a more reliable discriminator than IgG4 concentrations. Although IL-6 was not measured in patients with IgG4-RD, IL-6 elevations are known to be uncommon in IgG4-RD [23]. Therefore, the IgG4/IgG ratio and IL-6 may be a useful combination of laboratory tests for the discrimination of these two conditions.

Histologically, although both conditions are rich in plasma cells, several findings appeared to differ. In lymph nodes and lungs affected by iMCD, plasma cells were arranged in a sheet. This is endorsed as grade 3 plasmacytosis in the diagnostic criteria for iMCD. IgG4-RD was rich not only in plasma cells, but also large and small lymphocytes of variable maturation levels intermingled with plasma cells, creating a mixed lymphoplasmacytic appearance. This microscopic difference may represent the distinct underlying biology. In iMCD, hyper IL-6 is considered to be a strong inducer of B-cell maturation and immunoglobulin production [1, 24], while IgG4-RD is characterized by the expansion of not only plasma cells, but also other subsets of B cells (e.g., plasmablasts) [25, 26]. Regressed germinal centers in the lymph nodes were also characteristic of iMCD, but were less common than plasmacytosis. Similar to serological examinations, the IgG4/IgG-positive cell ratio is more relevant to the differential diagnosis than absolute IgG4-positive cell counts.

Sheet-like plasmacytosis was also observed in pulmonary manifestations of iMCD, indicating that the histologic part of the diagnostic criteria for iMCD is also applicable to extranodal organs. However, regressed germinal centers and follicular dendritic cell prominence may be less common at extranodal sites than in the lymph nodes. A characteristic feature in the lungs involved in iMCD was densely hyalinized fibrosis. This unusual finding may be equivalent to a hyaline vascular change in the lymph nodes, which is commonly observed in unicentric Castleman's disease. Storiform fibrosis and obliterative phlebitis were highly specific for IgG4-RD, but their incidences were not high in lung biopsies.

A small number of pathology studies previously compared iMCD and IgG4-RD [5, 6, 27]. IgG4-related lymphadenopathy is known to show various morphologic changes, including MCD-like (type I), follicular hyperplasia (type II), interfollicular expansion (type III), the progressive transformation of germinal centers (type IV), and inflammatory pseudotumor-like (type V) [27, 28]. Since type I cases may be misdiagnosed as iMCD, a careful clinicopathologic correlation and detailed microscopic analyses are crucial for these cases. Lung manifestations of iMCD were previously reported to show a lymphocytic interstitial pneumonia pattern [8, 9]. Terasaki et al. also recently compared the pathologic features of surgical lung biopsies between iMCD and IgG4-RD [5]. They examined patterns of lymphoplasmacytic infiltration and concluded that the perilymphatic infiltration of lymphocytes and plasma cells in IgG4-RD was more prominent than that in iMCD [5].

In 3 patients with iMCD, no lymphadenopathy was obvious on imaging at the initial presentation. Interestingly, 2 of them predominantly had lung abnormalities. Although the clinical presentation is atypical for iMCD, similar cases have been reported [7, 8]. These cases did not meet the diagnostic criteria for iMCD because lymphadenopathy at multiple sites is essential for the diagnosis [13]. It currently remains unclear whether they represent an extreme example of iMCD or a separate entity mimicking iMCD. We speculate that the former possibility is more likely given the histologic and serological similarities between the atypical cases and others with nodal manifestations. Characteristic serological findings including IL-6 elevations were observed in these cases, which also favors that possibility (Table 3) [8]. In addition, lymph node enlargement had become obvious in 2 cases during follow-up periods, leading to a more typical clinical appearance of iMCD. This clinical course suggests that lymphadenopathy may not be always present at the initial presentation; therefore, a diagnostic approach without lymph node biopsy may be required. Further studies will be needed in order to characterize those atypical cases.

Several previous studies examined potential cellular sources of IL-6 production in unicentric and multicentric Castleman's disease using *in situ* hybridization and immunohistochemistry [29–32]. In the 1990s, some investigators performed *in situ* hybridization for IL-6 mRNA on lymph node biopsies, and reported that follicular dendritic cells, lymphocytes, or macrophages were positive for IL-6 in MCD [30–32]. More recently, Post GR et al. reported that plasma cells and endothelial cells in the lymph nodes of iMCD were positive for IL-6 on immunohistochemistry [29]. However, to the best of our knowledge, no reliable antibody for IL-6 that works on formalin-fixed paraffin-embedded section is available. In addition, IL-6 expression in plasma cells is unexpected because plasmacytic differentiation is considered to be a result, but not an initiator, of hyper IL-6. In the present study, we used a novel highly sensitive protocol of *in situ* hybridization. Although one lung sample contained a positive focus, the remaining cases lacked IL-6 expression. The negative result was further confirmed by quantitative real-time PCR, which did not show a significant difference in the expression values of IL-6 mRNA among iMCD, IgG4-RD, and controls. Therefore, IL-6 is less likely to be produced locally in the lymph nodes or lungs. This potentially pathognomonic cytokine may be produced elsewhere and circulating IL-6 may lead to lymphoplasmacytosis in the affected organs. This unexpected finding requires future validation studies using other methods (e.g., frozen tissue, more sensitive assays).

In conclusion, although iMCD and IgG4-RD are both plasma cell-rich, multi-organ disorders, discriminating between the two will be possible, particularly based on age, affected organs, laboratory data (e.g., the IgG4/IgG ratio and IL-6), and microscopic findings (e.g., plasmacytosis, regressed germinal centers, densely collagenized fibrosis, eosinophils, and the IgG4/IgG-positive cell ratio). Serum IgG4 elevations and IgG4-positive cell counts appeared to be less useful than expected. The present results also suggested that IL-6 is not produced at sites affected by iMCD. IL-6 may be produced elsewhere, and circulating IL-6 may lead to lymphoplasmacytosis in the lymph nodes, lungs, and other organs.

## MATERIALS AND METHODS

### Comparison of clinicopathologic features

Clinical records were reviewed for age, sex, affected organs, and laboratory data including IgG, IgG4, CRP, and hemoglobin. IgG4 concentrations were not measured at the time of the diagnosis in 6 cases of iMCD. Serum IL-6 concentrations were available in 20 cases of iMCD. Affected organs were determined by imaging at the time of the diagnosis. Lymph node involvement was defined as the presence of enlarged lymph nodes (>1 cm in the short-axis diameter) according to the diagnostic criteria for iMCD [13].

Tissue specimens were fixed in 10% buffered formalin and embedded in paraffin. All samples were stained with hematoxylin and eosin (H&E), and lung biopsies underwent additional staining with Elastica van-Gieson (EVG). In lymph node biopsies, 5 individual histologic parameters described in the diagnostic criteria for iMCD were examined and graded into 0 to 3, with grades 2 and 3 being considered to be significant [13]. Samples were also reviewed in terms of the presence or absence of a broadened laminated mantle zone, and eosinophilic or neutrophilic infiltration (>10 cells/high power field [hpf]). Histopathologic findings examined in lung biopsies were the patterns of inflammatory extension (peribronchiolar, diffuse alveolar, or nodular [18]), types of infiltrating inflammatory cells, lymphoid follicle formation, types of fibrosis (either storiform or densely collagenized fibrosis), and the presence or absence of obliterative phlebitis.

Lymph nodes of iMCD were classified into three categories based on histopathologic changes according to the iMCD international diagnostic criteria [13]. Cases in which regressed germinal centers, follicular dendritic cell prominence, and hypervascularization were predominant features were called the hypervascular type, while cases with sheet-like (grade 3) plasmacytosis and hyperplastic germinal centers were the plasmacytic type. Cases with intermediate features were referred to as the mixed type [13].

### Immunohistochemistry

All cases were immunostained for IgG and IgG4 using a Ventana Benchmark XT (Ventana Medical Systems) according to the manufacturers’ protocols. Deparaffinized sections were heat-treated and incubated with primary antibodies. The antibodies used were as follows: IgG (clone RP023; prediluted; Diagnostic BioSystems, Pleasanton, CA) and IgG4 (clone HP6025; dilution 1:500; Invitrogen, Carlsbad, CA). The numbers of plasma cells positive for IgG or IgG4 were counted at three hot spots and calculated as the average per hpf. In endoscopic small lung biopsy specimens, positive cells were counted at the single highest spot.

### IL-6 mRNA *in situ* hybridization

*In situ* hybridization was conducted manually using RNAscope® (Advanced Cell Diagnostics, Newark, CA) in order to detect intracytoplasmic mRNA coding IL-6 according to the manufacturer's protocol. Briefly, 4-μm-thick sections of formalin-fixed paraffin-embedded tissue were baked and deparaffinized. After peroxidase blocking, antigen retrieval, and a protease treatment, slides were hybridized with the pre-designed probe specific for IL-6 mRNA (Advanced Cell Diagnostics). Signals were amplified by sequential hybridization and then visualized with Fast Red. Slides were counterstained with hematoxylin. Lung cancer tissue was used as a positive control.

### Quantitative real-time PCR

The expression of IL-6 mRNA in lymph node or lung biopsies was compared using real-time PCR among iMCD, IgG4-RD, and controls. Since the quality of extracted RNA was low or unstained slides were not available in some cases, this experiment was conducted on 26 lymph node samples (iMCD, n=11; IgG4-RD, n=10; disease controls, n=5) and 22 lung specimens (iMCD, n=4; IgG4-RD, n=8; disease controls, n=5; normal lung, n=5).

RNA was extracted from formalin-fixed paraffin-embedded tissue using the RNeasy FFPE Kit (Qiagen, Valencia, CA) following the manufacturer's instructions. Affected areas were selected for RNA extraction under a microscope. RNA content was assessed by the NanoDrop 2000c spectrophotometer (Thermo Fisher Scientific, Waltham, MA). cDNA was synthetized using the Super Script IV First-Stand Synthesis system (Invitrogen). Real-time PCR was performed for quantitative analyses, according to the standard protocol using a StepOnePlus Real-time PCR System (Applied Biosystems, Foster City, CA). Specific primers and probes for IL-6 (Hs00174131_m1) and β-actin (Hs01060665_g1) were obtained from Applied Biosystems. PCR was performed using the TaqMan Gene Expression Master Mix (Applied Biosystems) with 1 μl of cDNA in a 20-μl final reaction mixture. The mRNA expression levels of IL-6 were normalized to those of β-actin. Each experiment was performed in duplicate, and the mean was adopted for the analysis.

### Statistical analysis

Statistical analyses were performed using the Wilcoxon rank-sum test or Fisher's exact test for the comparison of clinicopathologic features between the two groups. In the analysis of real-time PCR, the Kruskal-Wallis test was applied to compare expression values among three or four groups, and if any significant difference was suggested, the Steel-Dwass test was conducted as a post-hoc analysis between the two relevant groups. A p-value less than 0.05 was considered to be significant. The software used for statistical analyses was JMP® 13 (SAS Institute Inc., Cary, NC, USA).

## Abbreviations

CRP: C reactive protein
HHV8: human herpes virus 8
hpf: high power field
IgG4-RD: IgG4-related disease
iMCD: idiopathic multicentric Castleman's disease
MCD: multicentric Castleman's disease.
